# Anti-VEGF Effect of Bioactive Indolic Compounds and Hydroxytyrosol Metabolites

**DOI:** 10.3390/foods11040526

**Published:** 2022-02-11

**Authors:** Marta Gallardo-Fernández, Ana B. Cerezo, Ruth Hornedo-Ortega, Ana M. Troncoso, M. Carmen Garcia-Parrilla

**Affiliations:** Departamento de Nutrición, Bromatología, Toxicología y Medicina Legal, Facultad de Farmacia, Universidad de Sevilla, C/P. García González No. 2, 41012 Sevilla, Spain; mgfernandez@us.es (M.G.-F.); rhornedo@us.es (R.H.-O.); amtroncoso@us.es (A.M.T.); mcparrilla@us.es (M.C.G.-P.)

**Keywords:** hydroxytyrosol, VEGF, eNOS, metabolites, 3,4-dihydroxyphenylacetaldehyde, indole pyruvic acid, angiogenesis

## Abstract

Angiogenesis is a key process involved in both cancer and cardiovascular diseases, the vascular endothelial growth factor (VEGF) and its VEGF receptor-2 (VEGFR-2) being the main triggers. The aim of this study was to determine the molecular mechanism underlying the potent inhibition of VEGF signaling by hydroxytyrosol (HT) metabolites and indolic compounds and establish a relation between their structure and bioactivity. Experiments involved the evaluation of their potential to inhibit VEGF on human umbilical vein endothelial cells (HUVECs) by ELISA assay and their subsequent effect on the downstream signaling pathway (PLCγ1, Akt, and endothelial nitric oxide synthetase (eNOS)) by Western blot. Respectively, 3,4-dihydroxyphenylacetaldehyde (DOPAL) (100 µM) and indole pyruvic acid (IPy) (1 mM) were capable of inhibiting VEGFR-2 activation with an IC_50_ value of 119 µM and 1.037 mM. The anti-angiogenic effect of DOPAL and IPy is mediated via PLCγ1. Additionally, DOPAL significantly increases eNOS phosphorylation, while IPy maintained it. These data provide for the first time evidence of the anti-angiogenic effect of DOPAL and IPy for future use as potential bioactive food ingredients.

## 1. Introduction

Several studies have described the association between the growth of malignant tumors and the development and growth of new blood growth vessels [1,2,3]. Angiogenesis is the physiological process in which new blood vessels are formed from pre-existing ones. VEGF is the most important player that regulates vessel formation during embryonic development, wound healing, and the maintenance of vessel homeostasis in adult organisms [4]. Angiogenesis in adults is involved in the development of cancer and cardiovascular diseases, favouring both tumor development and the development and destabilization of the atheroma plaque [3]. Indeed, tumors express various pro-angiogenic factors among them, the main one being VEGF which binds to VEGFR-2 [5]. VEGF can promote angiogenesis [2,6] exerting its angiogenic effects by stimulating VEGFR-2, which is critical to promote endothelial cell proliferation and differentiation [4,5]. Therefore, inhibiting angiogenesis by inhibiting the binding of VEGF to its receptor-2 is the mechanism that certain drugs use for the treatment of cancer and several other disorders, which is also a promising strategy for prevention through diet. In addition, it is worth noticing that certain anti-VEGF drugs, such as bevacizumab, sorafenib and sunitinib, cause hypertension as a side effect [7,8,9]. This is related to a nitric oxide (NO) reduction.

There is increasing interest in studying the anti-angiogenic properties of naturally occurring molecules in the diet. Additionally, *in vitro* studies have shown that compounds such as EGCG from green tea [10], procyanidin oligomers from apples [11] and resveratrol and quercetin found in grapes and red wine [12] possess anti-angiogenic effect. Furthermore, caffeic acid phenethyl ester derived from honeybee propolis [13], as well as urolithins [14] and ellagitannin [15] present in pomegranate and curcumin [16], have proven to exert anti-VEGF effect *in vivo*. More recently HT, which has been described both in fermented beverages and olive oil, as well as certain indolic compounds, such as 3-indolacetic acid (IAA) present in wine, have exhibited an IC_50_ against VEGFR-2 inhibition of 72.40 µM and 0.9704 mM, respectively [17,18]. Additionally, melatonin and serotonin simultaneously maintain eNOS phosphorylation, while EGCG, procyanidin tetramers and hydroxytyrosol increase it [10,17], which would avoid the adverse hypertensive effects associated with VEGF inhibitor drugs. This fact supports the advantage of these bioactive compounds over drugs.

The biological effect of HT largely depends on its bioavailability and metabolization, since for many polyphenols the formed metabolites present activity different from that of their parent compounds. HT is one of the major phenolic compounds in olive oil and table olives (5 mg of HT/20 g of olive oil) [19,20,21,22]. Furthermore, HT is present in wine in a range from 1.50 to 25 mg/L [23,24,25,26,27,28]. HT absorption occurs in the small intestine and colon [29] and reaches a maximum plasma concentration 5–10 min after ingestion [30]. Considering the bioavailability of HT (40–95%) [21,29,31] and plasma volume (5 L), the circulating HT would be between 0.15 and 37 µM [32]. Additionally, several studies have demonstrated that tyrosol is converted to HT *in vivo* [33]. Therefore, these findings imply that tyrosol intake also contributes to the circulating HT.

**Figure 1:**
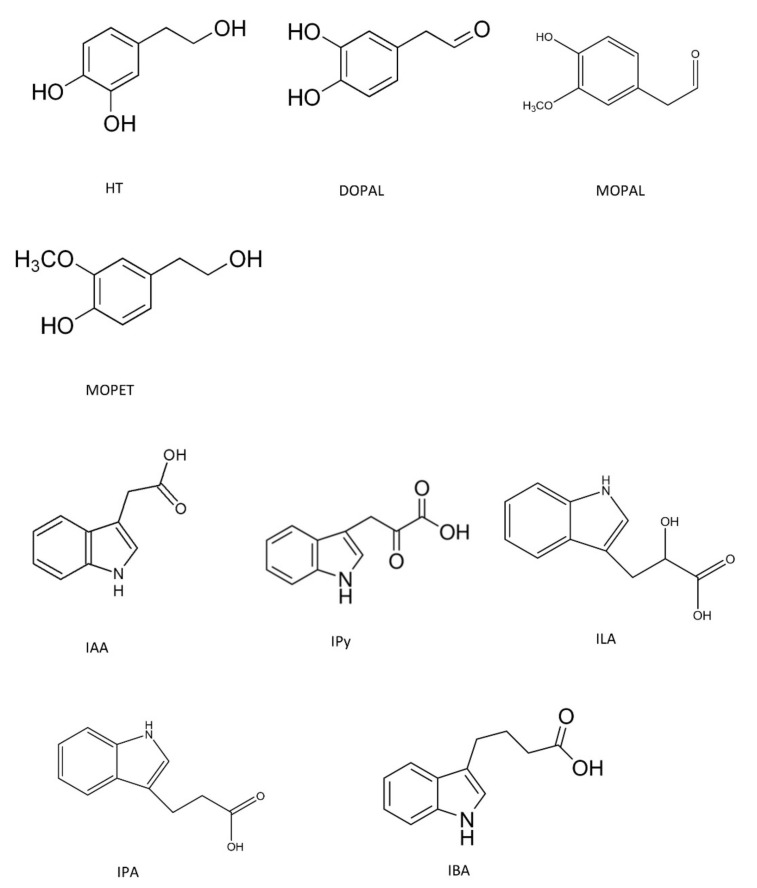
Chemical structure of HT metabolites and indolic compounds under study.

The low bioavailability of HT observed in various studies, related to the low concentrations of circulating HT, is due to phase I and II metabolism in the gut and liver. Enzymes involved in HT phase I metabolism, mostly present in the intestinal wall, are non-microsomal alcohol and aldehyde dehydrogenases, both located in the cytosol, resulting in metabolites such as DOPAL (Figure 1). DOPAL is not a specific metabolite of dietary HT since it is also a dopamine metabolite by deamination [34].

Furthermore, 4-hydroxy-3-methoxyphenylethanol (MOPET) is a methylated metabolite of HT (identified also in olive oil) [34] and 3-methoxy-4-hydroxyphenylacetaldehyde (MOPAL), the methylated metabolite of DOPAL. Additionally, MOPAL is part of dopamine metabolism [35,36]. Therefore, although DOPAL, MOPET and MOPAL are dietary metabolites of HT, their plasmatic levels are not specific to HT, since they also come from dopamine metabolism. In any case, in order to evaluate the overall effects of HT, there is a need to take into consideration the potential activity of its metabolites.

On the other hand, IAA is synthetized during alcoholic fermentation from tryptophan (L-TRP) amino acid by yeast [37]. Additionally, it was reported that about 3% of dietary L-TRP is converted to IAA in the gastrointestinal tract [38,39]. Some of the intermediaries of the IAA synthesis pathway with similar structure are IPy, indole propionic acid (IPA), indole butyric acid (IBA) and indole lactic acid (ILA) (Figure 1). These indolic compounds have been identified in fermented foods [40,41]. Additionally, IPy, IPA and ILA are human metabolites. IPy is synthesized endogenously in the intestinal microflora of mammals [42] and IPA and ILA have been described in plasma [43].

Concerning anti-angiogenic effects, nothing is known about the activity of the main human HT metabolites (DOPAL, MOPAL and MOPET) and of L-TRP metabolites (IPy, IPA, IBA and ILA). The original aim of the present study was to evaluate the anti-VEGF activity of HT metabolites and these four indolic compounds in order to establish a relation between structure and activity. Additionally, their intracellular mechanism of action was elucidated, showing their significant effect on eNOS activation and maintenance as a clear advantage over anti-VEGF drugs.

## 2. Materials and Methods

### 2.1. Chemicals and Reagents

DOPAL (Purity: ≥90%) was acquired from Cayman Chemical (Ann Arbor, MI, USA). MOPAL (99%), MOPET (99%), IPy (≥97%), IPA (≥99.0%), ILA (99%), IBA (≥99.0%), dimethyl sulfoxide (DMSO), and bicinchoninic acid (BCA) were obtained from Sigma–Aldrich (St. Louis, MO, USA).

HUVECs, endothelial cell growth medium-2 (EGM-2) and endothelial basal medium (EBM) were obtained from Lonza (Slough, UK). Recombinant human VEGFA_165_ was bought from R&D Systems (Minneapolis, MN, USA).

A PathScan^®^ Phospho-VEGFR-2 (Tyr1175) sandwich ELISA kit was purchased from Cell Signaling Technology (Hitchin, UK). NuPAGE lithium dodecyl sulfate (LDS) sample buffer and NuPAGE DTT and 4–12% Bis-Tris gels were obtained from Invitrogen (Loughborough, UK). Nitrocellulose 0.2 µm membranes was acquired from Bio-Rad (Hercules, CA, USA); p-PLCγ1, PLCγ1, p-Akt, Akt, p-eNOS, eNOS from Cell Signaling Technology (Danvers, MA, USA) and SuperSignal West Pico chemiluminescent substrate from Thermo Scientific (Hitchin, UK).

### 2.2. Cell Culture

HUVECs between passages 4 and 5 were grown in EGM-2.

### 2.3. Treatment of HUVECs

The HUVECs were grown to confluence and afterwards exposed to EBM including the individual compounds under study at the following concentrations: DOPAL, MOPAL and MOPET at 100 and 50 µM, and Ipy, IPA, ILA and IBA at 1 and 0.1 mM, agreeing with previous studies on molecules with similar chemical structure [17,18]. In all cases, the final concentration of DMSO was ≤0.1%. Furthermore, EBM (EGM-2, only including antibiotics, at ≤0.1% DMSO) was used for negative and positive vehicle controls (with and without 25 ng/mL of VEGF, respectively).

The cells were incubated with the individual compounds for 4 h at 37 °C. After that, HUVECs were stimulated with VEGF (25 ng/mL) for 5 min to determine VEGFR-2 phosphorylation, for 10 min to PLCγ1 phosphorylation, and for 60 min to Akt and eNOS phosphorylation. Every sample procedure was performed in duplicate, and experiments were carried out three times. HUVECs were then lysed with radioimmunoprecipitation assay (RIPA) buffer and subsequently centrifuged at 4 °C, 13,000 rpm for 10 min.

The protein content of the lysates was determined by the BCA assay. Furthermore, the DOPAL and the 3-indolpiruvic acid IC_50_ values were determined from five concentrations (180–50 µM and 0.7–2.0 mM, respectively).

### 2.4. Phosphorilated VEGFR-2 (ELISA Assay)

To measure the phosphorylated VEGFR-2 levels in the lysates, the sandwich ELISA kit for phospho-VEGFR-2 (Tyr1175) was used following the manufacturer’s instruction.

### 2.5. Western Blot Analysis for PLCγ1, Akt and eNOS

The protein lysates (26.8 μg) were mixed with NuPAGE lithium dodecyl sulfate (LDS) sample buffer, NuPAGE DTT, before heating for 10 min at 70 °C to denature proteins. Subsequently, electrophoresis was performed in NuPAGE 4–12% Bis-Tris gels before being transferred to 0.2 µm nitrocellulose membranes. Tris buffered saline with Tween 20 (TBST) was mixed with bovine serum albumin (BSA) to a final concentration of 5% (*w/v*) for blotting of the membranes before incubation with primary antibodies (p-PLCγ1, PLCγ1, p-Akt, Akt, p-eNOS, eNOS) overnight at 4 °C. Subsequently, secondary antibody (anti-rabbit IgG-HRP) in TBST + BSA (5%, *w/v*) was added to the membranes for 1 h at room temperature. 

The bands were visualized using a SuperSignal West Pico chemiluminescent substrate on an Amersham Imager 600 station (GE Healthcare live sciences, Marlborough, MA, USA). Samples were analysed in duplicate, and the assay was performed three times.

### 2.6. Statistical Analysis

Statistical analyses were carried out using Graphpad Prism software 8.0.2 (GraphPad Software, Inc., San Diego, CA, USA), using student’s *t* test to analyze significant differences between samples. The degree of significance of the analysis was as follows: *p* < 0.05 (*), *p* < 0.01 (**). Data are displayed as mean ± standard deviation.

## 3. Results

### 3.1. Anti-Angiogenic Effect by Inhibition of VEGFR-2 Activation in the Presence of Hydroxytyrosol Metabolites and Indolic Compounds

The first experimental design was carried out to evaluate the effect of the compounds under study against the activation of the VEGFR-2. Table 1 shows that MOPAL and MOPET (HT metabolites) did not inhibit VEGFR-2 activation. However, DOPAL at 100 µM was capable of inhibiting VEGFR-2 activation by 46%. Therefore, the IC_50_ value was tested by ELISA between 180 and 50 µM, showing an IC_50_ value of 119 µM.

Additionally, Table 1 shows that IPy was the only indolic compound inhibiting VEGF induced VEGFR-2 activation by 4% and 56% at 0.1 and 1 mM, respectively. Secondly, the pre-incubation treatment with the IPy (0.7–2.0 mM) resulted in an IC_50_ value of 1.037 mM.

### 3.2. Effects of DOPAL and 3-Indolpiruvic Acid on PLCγ1, Akt and eNOS

Since DOPAL and IPy acid were shown to inhibit VEGF-induced VEGFR-2 phosphorylation, we evaluated whether they are able to regulate downstream signaling events of p-VEGFR-2. We assessed their effect in PLCγ1, the main protein involved in the cell proliferation, as well as Akt and eNOS, proteins downstream activated in the VEGF signaling and involved in the vasodilation.

**Figure 2:**
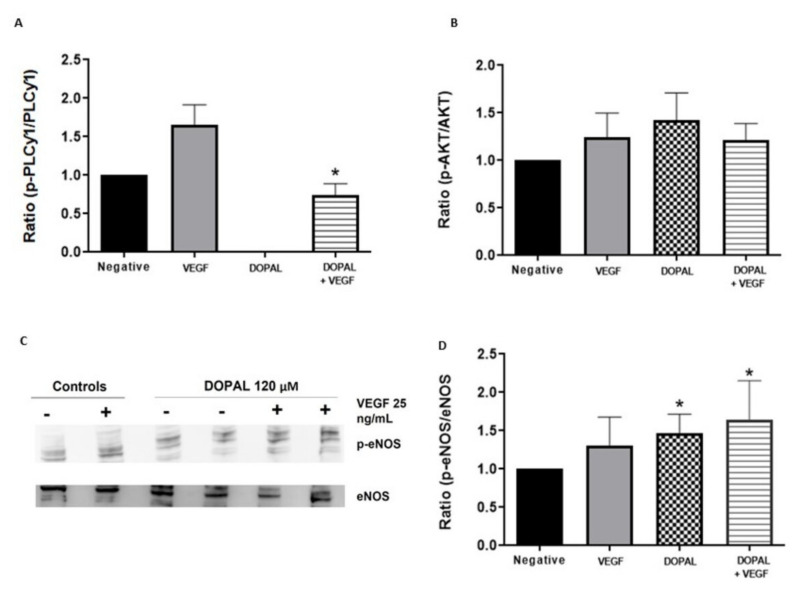
Effects of DOPAL on PLCγ1, Akt and eNOS. HUVEC cells were treated with DOPAL (120 µM) for 4 h and then stimulated with VEGF (25 ng/mL) for 10 min (**A**) and 60 min (**B**–**D**). Western-blot membranes were incubated with anti PLCγ-1 and anti p-PLCγ-1 (**A**), anti Akt and anti p-Akt (**B**) and anti eNOS and anti p-eNOS (**C**,**D**) antibodies. Data representation of p-PLCγ-1/PLCγ-1, p-Akt/Akt and p-eNOS/eNOS ratio are displayed as mean ± SD (*n* = 5). * *p* < 0.05 against VEGF alone (**A**) and versus negative control (**D**).

**Figure 3:**
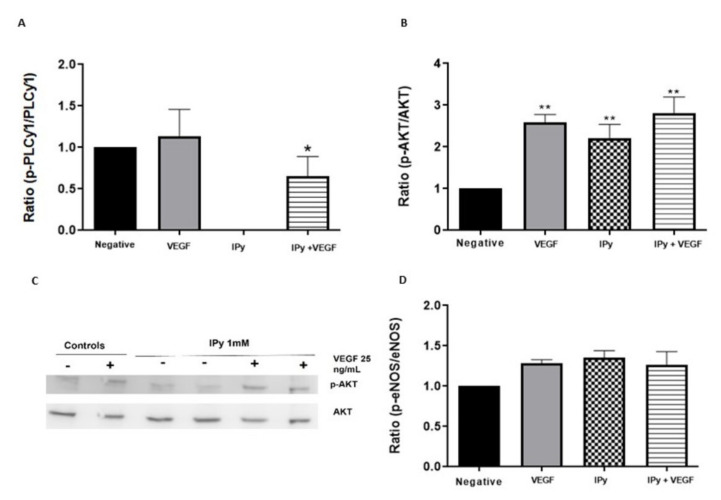
Indole pyruvic acid inhibits PLCγ1 phosphorylation while activate Akt and eNOS. HUVEC cells were treated with IPy (1 mM) for 4 h and then stimulated with VEGF (25 ng/mL) for 10 min (**A**) and 60 min (**B**–**D**). Western-blot membranes were incubated with anti PLCγ-1 and anti p-PLCγ-1 (**A**), anti Akt and anti p-Akt (**B**,**C**) and anti eNOS and anti p-eNOS (**D**) antibodies. Data representation of phosphorylated antibody/total antibody ratio is indicated as mean ± SD (*n* = 5). * *p* < 0.05, ** *p* < 0.01 against VEGF alone (**A**) and versus negative control (**B**).

Figure 2A and Figure 3A showed that DOPAL and IPy caused a significant decrease in the pPLCγ1/PLCγ1 ratio in comparison with the positive control (VEGF only). These data demonstrate that these compounds are not only inhibiting VEGFR-2 activation, but they are also preventing downstream signaling through PLCγ1.

Additionally, DOPAL maintained the activation of Akt both in the presence and absence of VEGF (Figure 2B). Subsequently, Figure 2C,D show that DOPAL significantly increases the p-eNOS/eNOS ratio in the presence and absence of VEGF stimulation. These results are reliable since they indicate that DOPAL is able to inhibit the angiogenesis activated by VEGF, while increasing the activation of eNOS, which is a potent vasodilator.

IPy significantly increased Akt activation in the presence and absence of VEGF (Figure 3B). Figure 3D presents the western blot bands, in which the activation of AKT by IPy is observed in the presence and absence of VEGF. Consequently, the p-eNOS/eNOS ratio is also maintained by IPy in the presence and absence of VEGF, as shown in Figure 3C.

## 4. Discussion

In order to understand how diet can prevent angiogenic related diseases, it is necessary to know the underlying molecular mechanism that explains the effect of the bioactive compounds naturally present in foods and beverages as well as their derived metabolites. In previous studies, the anti-angiogenic effect of compounds derived either from tyrosine, such as HT [17], or from L-TRP, such as IAA [18], has been highlighted. Now, we explore the bioactivity of HT endogenous metabolites such as DOPAL, MOPAL and MOPET and other L-TRP metabolites such as IPy, IPA, ILA and IBA.

**Figure 4:**
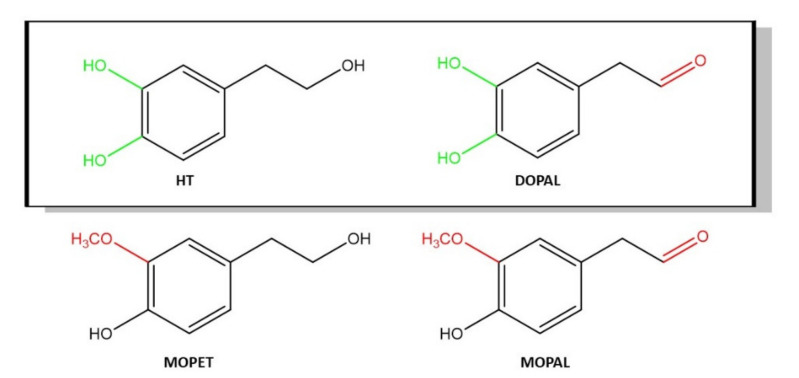
Chemical characteristics of HT and its metabolites which seem to drive VEGF inhibition (green) and non-effect (red).

In the present study, we present for the first time evidence that DOPAL possesses anti-angiogenic activity (IC_50_: 119 µM), while MOPAL and MOPET were inactive. DOPAL, MOPAL and MOPET have in common that they present a benzene ring (Figure 1). One of the differences between them is the type of substituents attached to this benzene ring. DOPAL possess a catechol group while MOPAL and MOPET present a substitution of one methoxy group instead of one of the hydroxyl groups. If the ring is attached to a group that donates electrons, such as the -OH group, the electron density of benzene will be higher, and its reactivity will increase. In fact, the catechol group was one of the chemical characteristics strongly related to a potent VEGF inhibition [11]. This might explain why DOPAL is the only compound of the three HT metabolites under study that has anti-VEGF activity (Figure 4), similarly to HT (IC_50_: 72 µM) [17]. However, MOPAL and MOPET present a substitution of the hydroxyl group with a methoxy group, which results in a lower reactivity. These results agree with previous studies in which methylation of one of the hydroxyl groups of the catechol, on the B-ring, of quercetin to turn it into tamarixetin caused a loss in its activity against the inhibition of VEGFR-2 [11]. In addition, HT (IC_50_: 72 µM) [17] has higher anti-angiogenic activity than DOPAL (IC_50_: 119 µM). It seems that the functional group (alcohol or aldehyde) produces a shift in the activity of the molecule (Figure 4).

Furthermore, if we compare these results with those previously reported for certain bio-actives such as the flavonoids, quercetin, quercetagetin, luteolin, and orobol, they all display higher anti-VEGF activity (IC_50_ = 0.754, 0.096, 7.46, 3.311, respectively) [11] than DOPAL. Although they all present a catechol group, it seems that other chemical characteristics such as the C6–C3–C6 structure, the C2═C3 double bound in the C-ring, the 4-oxo group in the C-ring, or even the molecular size present in the former, are required for additional anti-VEGF activity.

Plasma concentration of DOPAL has been previously reported at 9.75 pg/mL, which is equal to 6.4 × 10^−5^ µM [44]. This concentration is quite far from its IC_50_. Noteworthy, Burke et al. [44] obtained the plasma sample from a healthy male from a Missouri population who did not follow a controlled diet. Therefore, further controlled study in a population following a Mediterranean diet rich in HT such as olive oil should be carried out in order to determine this metabolite concentration in plasma.

It has already been demonstrated that certain indole compounds such as melatonin (MEL), serotonin (SER) and IAA inhibit VEGFR-2 phosphorylation [15]. IAA was the compound that displayed the greatest anti-angiogenic effect with a percentage of inhibition of VEGFR-2 activation of 54% at a concentration of 1 mM (IC_50_: 0.97 mM). In the present study, we tested indolic compounds with a similar structure to IAA (IPy, IPA, ILA and IBA) to evaluate their effect against VEGFR-2. The only compound under study with activity against VEGFR-2 activation was indole pyruvic acid with an IC_50_ of 1.037 mM, similarly to that previously described for 3-indole acetic acid (IC_50_ was 0.9 mM) [18]. If we compare the structure-activity, all indolic compounds under study and IAA have the same indole ring in common. However, the side chain differs when comparing them. Our results demonstrated that an increase in the chain length as in the case of IPA and IBA (Figure 1) does not produce activity against the inhibition of VEGFR-2 activation. If the chain is elongated and has a hydroxyl substituent, it also has no activity as occurs with ILA. However, in the case of IPy, in which the chain increases but has a keto group, the activity is maintained. This is probably because the double bond promotes movement of electrons and, consequently, its reactivity and activity are maintained. Additionally, our results shows that IPy possess higher anti-VEGF activity than melatonin > serotonin > 5-hydroxytryptophol at 1 mM concentration (32%, 30%, and 23% of inhibition, respectively) [18].

The relationship between structure and anti-angiogenic activity of phenolic compounds has been widely studied since we know that the catechol group confers functionality to the benzene ring and therefore increases the bioactivity of the molecule [11]. However, so far, the relationship between structure and activity of indolic compounds has not been evaluated. This study contributes to a first approach towards further prediction of the functionality of indolic compounds and non-flavonoid polyphenols, which would help to understand how microorganism’s metabolites could present a bioactive role. Further evaluation of the anti-VEGF activity of more indolic and phenolic compounds with different structures would be needed to stablish a proper quantitative structure–activity relationship (QSAR) model [45]. VEGF-induced VEGFR-2 activation triggers a complex intracellular signaling cascade regulating a wide range of endogenous molecules involved in angiogenic signal transduction. DOPAL and IPy have been demonstrated to inhibit VEGF-induced VEGFR-2 phosphorylation, so we have evaluated their effect on the signaling cascade.

Firstly, we have evaluated the effect of DOPAL and indole pyruvic in regulating both migration and cell proliferation by studying the activation of PLCγ1, the first constituent of the main VEGFR-2 pathway. The results showed that, after VEGF stimulation, PLCγ1 became phosphorylated. However, pre-incubating the cells with DOPAL and IPy caused significant decrease in the pPLCγ1/PLCγ1 ratio compared to the positive control with only VEGF (Figure 2 and Figure 3).

Inhibition of PLCγ1 activation provides information on the mechanism of action of compounds with an anti-angiogenic effect. After evaluating the results obtained, in the case of DOPAL and IPy their anti-angiogenic mechanism could be proposed by means of PLCγ1. The ability to inhibit the PLCγ1 phosphorylation of DOPAL agrees with the results described in the literature, which confirmed that the anti-angiogenic effect of HT is also mediated by PLCγ1 inhibition [17]. However, previous studies have shown that the anti-angiogenic effect of certain indolic compounds such as IAA is not mediated by PLCγ1 inhibition [17]. Present results, therefore, demonstrate for the first time that the anti-angiogenic effect of DOPAL and IPy is regulated by inhibition of PLCγ1 phosphorylation. 

Vasodilation is also stimulated through VEGF-induced VEGFR-2 activation. This activates eNOS, by means of Akt, triggering the production of NO [46]. Hypertension is a common side effect of anti-VEGF therapies such as sorafenib [9], sunitinib [47], pazopanib [48] and axitinib [49]. However, the present study has proven that DOPAL can significantly increase the phosphorylation of eNOS while simultaneously inhibiting VEGFR-2 activation. These results agree with other bio-actives such as procyanidins dp4, EGCG and HT which have proven to activate eNOS via Akt [10,17]. Additionally, IPy maintains the phosphorylation of eNOS, agreeing with the melatonin and serotonin effect on eNOS [17]. Therefore, DOPAL and IPy may be expected to stimulate NO bioavailability preventing the adverse side effects related to VEGF inhibitors. Future studies are required to evaluate the *in vitro* NO increase induced by DOPAL and IPy, related to a vasodilation effect, as a clear advantage over anti-VEGF drugs. Moreover, to reinforce their anti-angiogenic effect, further studies regarding inhibition of migration (*in vitro* wound-healing assay) and new blood vessels formation (*ex vivo* aortic rings model) would be needed.

## 5. Conclusions

The present data support the notion that certain specific bio-actives are able to inhibit VEGF-induced VEGF-2 activation, in which the chemical structure plays a crucial role. DOPAL and IPy presented an IC_50_ value of 119 µM and 1.037 mM, respectively. The orto--position of the -OH groups in the HT metabolite DOPAL explains its inhibitory effect, while the methylation of one of the OH renders the molecules inactive (MOPET and MOPAL). Additionally, the oxidation of the -OH group to an aldehyde group in the chain attached to the aromatic ring decrease the bioactivity. Furthermore, elongation and substituents in the chain of the indolic compounds are crucial for their bioactivity, the keto group substituent being the most active (IPy). The present results demonstrate for the first time that the anti-angiogenic effect of DOPAL and IPy is regulated by inhibition of PLCƴ1 phosphorylation, while they activate and maintain eNOS phosphorylation (a potent vasodilator), respectively, via Akt. DOPAL and IPy are potential compounds for future use as anti-VEGF ingredients.

## Figures and Tables

**Table 1:** Inhibition of VEGF-induced VEGFR-2 phosphorylation and IC_50_ values of HT metabolites and indolic compounds.

Compounds	Concentrations	Inhibition (%)	IC_50_
DOPAL	50 µM	5 ± 7	119 µM
	100 µM	46 ± 8	(95.49–148.1)
MOPAL	100 µM	NI	ND
MOPET	100 µM	NI	ND
IPy	0.1 mM	4 ± 10	1.037 mM
	1 mM	56 ± 0.7	(0.9061–1.171)
IPA	0.1 mM	NI	ND
	1 mM	NI	
ILA	0.1 mM	NI	ND
	1 mM	NI	
IBA	0.1 mM	NI	ND
	1 mM	NI	

Inhibition percentages of VEGF-induced VEGFR-2 activation are expressed as the mean ± SD (*n* = 3). The 95% confident intervals are shown in brackets. NI: non-inhibition. ND (not determined). DOPAL (3,4-dihydroxyphenylacetaldehyde); MOPAL (3-methoxy-4- hydroxyphenylacetaldehyde); MOPET (4-hydroxy-3-methoxyphenylethanol); IPy (indole pyruvic acid); IPA (indole propionic acid); indole lactic acid (ILA); IBA (indole butyric acid).

## Data Availability

The datasets generated for this study are available on request to the corresponding author.
